# A simplified strategy for titrating gene expression reveals new relationships between genotype, environment, and bacterial growth

**DOI:** 10.1093/nar/gkaa1073

**Published:** 2020-11-22

**Authors:** Andrew D Mathis, Ryan M Otto, Kimberly A Reynolds

**Affiliations:** The Green Center for Systems Biology, The University of Texas Southwestern Medical Center, Dallas, TX 75390, USA; The Green Center for Systems Biology, The University of Texas Southwestern Medical Center, Dallas, TX 75390, USA; The Green Center for Systems Biology, The University of Texas Southwestern Medical Center, Dallas, TX 75390, USA; Department of Biophysics, The University of Texas Southwestern Medical Center, Dallas, TX 75390, USA

## Abstract

A lack of high-throughput techniques for making titrated, gene-specific changes in expression limits our understanding of the relationship between gene expression and cell phenotype. Here, we present a generalizable approach for quantifying growth rate as a function of titrated changes in gene expression level. The approach works by performing CRISPRi with a series of mutated single guide RNAs (sgRNAs) that modulate gene expression. To evaluate sgRNA mutation strategies, we constructed a library of 5927 sgRNAs targeting 88 genes in *Escherichia coli* MG1655 and measured the effects on growth rate. We found that a compounding mutational strategy, through which mutations are incrementally added to the sgRNA, presented a straightforward way to generate a monotonic and gradated relationship between mutation number and growth rate effect. We also implemented molecular barcoding to detect and correct for mutations that ‘escape’ the CRISPRi targeting machinery; this strategy unmasked deleterious growth rate effects obscured by the standard approach of ignoring escapers. Finally, we performed controlled environmental variations and observed that many gene-by-environment interactions go completely undetected at the limit of maximum knockdown, but instead manifest at intermediate expression perturbation strengths. Overall, our work provides an experimental platform for quantifying the phenotypic response to gene expression variation.

## INTRODUCTION

Gene expression changes provide a critical mechanism by which cells adapt to environmental challenges, evolve new metabolic function, and regulate growth rate. The relationship between gene expression level and cellular growth rate is complex: it is often nonlinear, sometimes non-monotonic, and depends on both the environmental context and genetic background (1–5). Nonetheless, many genome-scale screens compress this complexity into a single perturbation per gene, often at the limit of extreme knockdown or complete knockout (6–11). This methodological limitation obscures the expression-dependency of genetic and environmental interactions. It also reduces the utility of high-throughput screening data in constructing and testing quantitative models of cell growth rate. Development of new tools for high-throughput titration of gene expression and precise quantification of the corresponding effects on growth rate is essential to study, model, and engineer this fundamental relationship.

Existing methods for titrating gene expression have provided many insights but are limited in both throughput and generality. Chemically inducible promoters, mutated promoter libraries, and alternative ribosomal binding site libraries have all been used to gradate gene expression or downstream protein abundance (2,12–16). However, these methods require either moving the genes of interest to a plasmid, or chromosomal insertion of the promoter or RBS upstream of the gene of interest. This consequently limits throughput. Moreover, many of these approaches are difficult to extend to combinations of genes (e.g. to measure pairwise or higher-order genetic interactions), and not all are amenable to high-throughput measurements of growth rate by next generation sequencing.

Interestingly, recent work has shown that modulation of gene expression can be achieved using CRISPR interference (CRISPRi) (17–20). The CRISPRi system uses single guide RNAs (sgRNAs) and a catalytically dead DNA endonuclease (dCas9) to target and transcriptionally repress specific genes (21,22). Bikard and colleagues showed that adding a series of mutations to the CRISPRi sgRNA homology region can titrate the expression of chromosomally encoded fluorescent proteins, seemingly by tuning the rate at which RNA polymerase ‘kicks out’ the bound dCas9 (17,18). In contrast to other approaches for titrating gene expression, CRISPRi can be used in any model system that will express the dCas9 and sgRNA constructs. By sequencing the sgRNA array—a linear stretch of a few hundred base pairs—we can determine the gene(s) targeted for knockdown. As a consequence, next generation sequencing can be used to track the relative frequency (and thus growth rate) of each sgRNA variant in libraries of 10–10^4^ distinct knockdowns. This approach is sometimes referred to as CRISPRiSeq (23–25).

In this work, we combined titratable CRISPRi sgRNAs with CRISPRiSeq to create a general, high throughput approach for titrating gene expression and quantifying the effects on *Escherichia coli* bacterial growth rate. Through the testing of multiple sgRNA library designs, we defined a straightforward mutation strategy that produces near-monotonic titrations of growth rate effect while maintaining a compact library size. Our method incorporates a molecular barcoding approach for detecting CRISPRi ‘escapers’—mutated cell populations that evade the CRISPRi machinery. With this approach, we quantified the growth rate effects of CRISPRi titration curves in both glucose and glycerol carbon sources under turbidostat growth conditions. We found 37% more gene-by-environment interactions using CRISPRi titration curves than with the standard approach of a single (maximal-strength) knockdown per gene. Taken together, our work provides a straightforward experimental strategy to quantitatively characterize growth rate dependencies on gene knockdown, and reveals a more complete picture of gene-by-environment interactions.

## MATERIALS AND METHODS

### Gene selection for titrated expression perturbations

**Figure 1.**
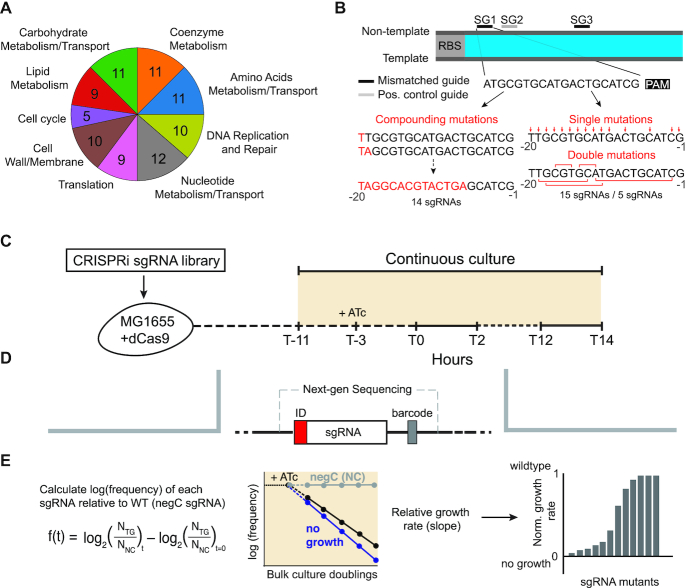
An overview of the titratable CRISPRi experiment. (**A**) Target genes for CRISPRi were selected from nine different cellular processes as defined by Clusters of Orthologous Groups functional annotations (28). (**B**) Three parent sgRNAs were designed per gene (labeled SG1, SG2, SG3). SG1 and SG2 were designed to be as close as possible to the translation start site without targeting overlapping sequences. SG3 was designed to be at least 200 base pairs downstream. SG1 and SG3 were then mutated according to three different mutational strategies: compounding, single or double mutations. Red indicates selected mutation sites. All mutations were made to the complementary base pair. (**C**) The CRISPRiSeq growth rate assay. The CRISPRi sgRNA plasmid library was transformed into MG1655 *E. coli* with a chromosomally-encoded, anhydrotetracycline (ATc) inducible dCas9. After transformation and overnight growth, cells were washed into minimal media and adapted to turbidostat conditions. ATc was added to induce CRISPRi, and culture time points were collected every two hours for next-generation sequencing. (**D**) To measure sgRNA frequencies over time, PCR was used to amplify the sgRNA containing region of the expression vector and add adaptors for NGS. This region includes a six nucleotide barcode that is used to track internal replicates and detect constructs with anomalous growth. (**E**) Frequency was calculated relative to a non-targeting (negC) sgRNA and time point zero hours. We performed a linear fit for the change in log(relative frequency) over time for each sgRNA relative to the negC sgRNA. The slope of this fit was relative growth rate. Relative growth rates were rescaled so that negC (wildtype-like growth) is 1 and no growth is 0 (methods).

We selected 88 genes from diverse cellular processes as a test set for developing and evaluating our approach (Figure 1A and Supplementary Table S1). Of these genes, 13 were selected from one-carbon folate metabolism and 11 genes were selected from glycolysis. The remaining 64 genes were selected based on the following criteria: (i) the gene was required for growth in MOPS minimal media containing glucose according to (26) (either the knockout cannot be made, or the knockout does not surpass an optical density at 600 nm (OD_600_) of 0.01 over 24 h), (ii) the gene product had an estimated copy number per cell based on previous work by Schmidt *et al.* (27), (iii) the gene was annotated in the bacterial Clusters of Orthologous Genes (COGs) Database (28), and (iv) the gene had a chromosomal YFP-fusion available (29). Ninety-six *E. coli* genes met these criteria. This list was further reduced to 64 genes by (i) removing any genes that were previously selected from glycolysis and folate metabolism and (ii) subsampling from nine diverse COG defined biological process: carbohydrate metabolism and transport, coenzyme metabolism, amino acids metabolism and transport, DNA replication and repair, nucleotide metabolism and transport, translation, cell wall and membrane, cell cycle, and lipid metabolism.

### CRISPRi sgRNA library design

For each of the 88 genes, we attempted to design three parent sgRNAs (referred to as SG1, SG2, and SG3, Figure 1B). Each parent sgRNA contained a twenty base pair targeting region that was 100% homologous to the non-template strand of the target gene and adjacent to a Protospacer adjacent motif (PAM) site of motif CCN. SG1 and SG2 were selected to be located within the first 150 bp of the gene and as close to the translation start site as possible without overlapping. SG3 was selected to be at least 200 base pairs from the translation start site but as close to the 200 base pair mark as possible. All parent sgRNAs had to meet the following criteria: (i) >45% GC content and <80% GC content, (ii) no poly-T repeat longer than four, and (iii) low off target binding potential as determined by BLASTn.

All parent sgRNAs were checked for potential off-target binding by BLASTn against the *E. coli* MG1655 genome (taxid:511145). Parent sgRNAs with more than 75% homology to another chromosomal location were further analyzed for off target potential. If any of the following criteria were met, the sgRNA was redesigned: (i) the sgRNA had 100% homology to more than one place in the chromosome; (ii) the sgRNA had greater than 90% homology to the off target binding location and was adjacent to a PAM site; (iii) the sgRNA had >75% homology but <90% homology to an off-target binding site, region 1 of the sgRNA (the eight base pairs immediately proximal to the PAM binding site and most sensitive to mutation) had no mismatches, and the off target binding site was adjacent to a PAM site. For 78 of the 88 genes, three viable parent sgRNA could be designed within these constraints and all genes except *nadE*, *pykF*, and *pyrH* had at least two parent sgRNAs designed.

From this set of parent sgRNAs, 31–66 mutated sgRNAs were designed to titrate the expression of each gene. (Supplementary Table S1). To achieve this, parent sgRNAs SG1 and SG3 were mutated in three different ways (Figure 1B). First, we chose single positions to mutate based on prior findings that mutations closer to the PAM site (in the ‘seed region’) have severe effects on knockdown strength and those more distal exhibit titrated effects on knockdown (17,20). As such, positions −1, −2, −5, −8, and −10 through −20 where all mutated individually. Second, we used a strategy by Bikard *et al.* that showed that serially adding a single mutations at position −20 and proceeding with single mismatches to position −7 also had gradated effects on fluorescent protein expression (17,18). These are called compounding mutations. Lastly, we chose to include five double mismatches at positions −2/−12, −12/−14, −15/−17, −11/−18, and −13/−19. Mutations were always made to the complement base. SG2 was not mutated but was used as a separate control for CRISPRi knockdown, under the expectation that SG1 and SG2 should show similar growth rate effects. Negative control sgRNAs expected to have little effect on gene expression were also included in the library (Supplementary Table S1). These comprise 45 sgRNAs with random 20 bp homology regions.

### sgRNA library construction and assembly

All sgRNAs were synthesized by Twist Biosciences as an oligo pool. Synthesized sgRNA oligos contained a promoter, homology region, loop, and terminator as described by Qi *et al.* (20,21). The oligo pool was amplified using primerGG_F and primerGG_R according to the standard protocol from Twist Biosciences using Kapa DNA polymerase (Supplementary Table S2, Thermo, #NC0636151). Initial denaturation was at 95°C for 3 min, amplification denaturation was at 98°C for 20 s, PCR annealing was at 58°C for 15 s, elongation was at 72°C for 15 s, and final elongation was at 72°C for 1 min. Eleven PCR amplification cycles were performed. The primers added flanking BsaI sites that were used to insert the sgRNA library into six low copy number expression plasmids. These plasmids were modified versions of the pCRISPR plasmid (Addgene #42875, (30)) constructed by removing the original gRNA spacer sequences by restriction digest and replacing them with a small DNA sequence containing two BsaI sites for golden gate cloning. We also added a 6 bp DNA barcode downstream of the sgRNA on the plasmid, to allow for internal replicate measurements during the experiment. The 6N barcode sequences were CTTTCA, ATCATG, GCATGG, GTATGA, AGTCTA, and CCTAGT. The complete sgRNA library was inserted into all six expression vectors by Golden Gate Cloning to yield six internal replicate populations.

Golden Gate Cloning was performed as described by Hawkins *et al.* with a few adjustments (21). Each reaction consisted of 75 ng of expression vector, 6 ng of amplified sgRNA library insert, 2 μl T4 10× ligase buffer, 1 μl T4 ligase (2000 U/μl, NEB, #M0202M), 1 μl BsaI (NEB, #R0535S), and MB H_2_O to 20 μl. The Golden Gate reaction was performed for 25 repeated cycles of 37°C (2 min), 16°C (3 min) in the 2720 Thermal Cycler (Applied Biosystems). The reaction was completed at 50°C for 10 min followed by 80°C for 20 min. Negative control reactions had sgRNA library inserts with BsaI sites that were not compatible with complete Golden Gate assembly, and therefore resulted in linear DNA fragments.

Golden gate reactions were cleaned up using the Zymo Clean & Concentrate kit (Zymo Research, #D4014), and 1 μl of each reaction was transformed into XL1 Blue electrocompetent cells. Transformations were recovered in SOB for 1 h at 37°C shaking 220 rpm. 10 μl of transformant was diluted 1/10 in SOB and 50 μl was plated on LB 35 μg/ml Kanamycin plates to estimate efficiency. The remainder of the transformation was back diluted using LB, Kanamycin was added to 35 μg/ml, and grown overnight at 37°C shaking. Total colony forming units for each expression vector was at least 7.5-fold greater than library size suggesting good library representation. Plasmids from overnight cultures were purified using a Gene-Jet kit (Thermo, K0503) and different barcoded expression vectors were combined in equimolar ratio. The assembled library was transformed (as above) into electrocompetent MG1655 *E. coli* with dCas9 in the HK022 attB site (a gift from the Bikard lab) hence referred to as gAM-513. Total CFU was 36.8 million which is 6000× above total library size.

### Pooled library growth rate assay and next generation sequencing

After transformation, the gAM-513 overnight culture was washed 2 times with 1 ml M9 minimal media pH 6.5, 0.4% glucose, and 35 μg/ml Kanamycin (hence referred to as M9-glucose). gAM-513 was resuspended into 1 ml M9-glucose, back diluted 1:50 into 8 ml M9-glucose, and grown at 37°C shaking. After 12 h of outgrowth and adaptation to M9 media, the culture was back diluted to OD_600_ = 0.05 and grown in the turbidostat for an adaptation period of 8 h (Figure 1C). Our turbidostat was constructed in house following the design of Toprak *et al.* (31,32). The turbidostat clamped optical density to OD_600_ = 0.15, with temperature set to 37°C throughout the experiment. After turbidostat adaptation, 50 ng/ml anhydrotetracycline (ATc) was added to induce CRISPRi expression. After 3 h of ATc induction, a 1 mL culture time point was taken, spun down (3000 × g, 5 min), decanted, and the pellet was stored at −20°C for downstream sequencing (time point 0 hours). Time points were taken every two hours for 14 additional hours (with the exception of the 8 hour time point, which was omitted), and processed and stored in a similar manner (Figure 1C). The experiment in glycerol was conducted identically, except that 0.4% glucose was replaced with 0.2% glycerol as the carbon source in the M9 minimal media.

For each time point, the sgRNA containing region was amplified by PCR and deep sequenced (Figure 1D). DNA was extracted from time points by addition of 100 μl MB H_2_O, lysis at 95°C for 3 min, centrifugation at 20 000 × g to remove lysate, and decanting into a new eppendorf tube. sgRNA regions where amplified using custom TruSeq_F and TruSeq_R primers (Supplementary Table S2). Amplification was performed using Q5 high fidelity hot-start polymerase (NEB # M0493L) in the following master mix optimized for specific amplification of target fragment: 5 μl 10× Q5 buffer, 5 μl 50% glycerol, 0.5 μl 10 mM dNTPs, 1.25 μl 10 μM F primer, 1.25 μl 10 μM R primer, 10.5 μl MB H_2_O, 1 μl template, and 0.5 μl Q5 polymerase (Supplementary Table S2). The PCR was run for 7 cycles under standard conditions with annealing at 61°C. TruSeq PCR reactions were then amplified using i5/i7 primers to provide unique sequencing indices to each time point. The i5/i7 reaction was performed under identical conditions for 20 cycles. Amplified DNA from i5/i7 reaction was quantified using the picogreen assay (Thermo #P7589) and then time points were mixed in equal ratio. The mixed time points were gel purified in a 1% agarose TAE gel with EtBr as the stain, and the DNA was purified using the Zymo Gel DNA Recovery Kit (Zymo, #D4008). DNA quality was determined by 260/230 and 260/280 nanodrop ratios and quantified using the Qubit 3 (Thermo). Quantified DNA was sent to GeneWiz for Illumina HiSeq Sequencing, using a 300 cycle paired end run.

### Fitting relative growth rates

Paired end reads contained in fastq files were first merged using USEARCH v11 (33,34). Then, custom Python2.7 analysis code was used to process the resulting fastq files (available on github: https://github.com/reynoldsk/titratableCRISPRi). For every time point, the presence of each unique sgRNA was counted (Figure 1E, Supplementary Tables S3 and S4). In order to be counted, the sgRNA binding region sequence had to: (i) exactly match a sequence in the designed library and (ii) each base pair in the sequence had to have a *Q*-score > 30 (*P*-value < 0.001). In additional to being counted by sgRNA identity, reads were further subdivided by barcode into six replicate populations. Here, and throughout the manuscript, we refer to these six replicate populations as internal replicates.

Allele frequency was calculated by normalizing sgRNA (*n*_sg_) counts by negative control (non-targeting sgRNA) counts (n_nc_) at each time point (*t*) and at the first time point (*t* = 0). The negC sgRNA used for normalization was selected from the center of the scrambled negC growth sgRNA distribution. This was negC_rand_42.

$$\begin{equation*}{\rm frequency}\;\left( f \right) = {\log_2}{\left( {\frac{{{n_{{\rm sg}}}}}{{{n_{{\rm nc}}}}}} \right)_t} - {\log_2}{\left( {\frac{{{n_{{\rm sg}}}}}{{{n_{{\rm nc}}}}}} \right)_{t = 0}}\end{equation*}$$

Relative growth rates were calculated by fitting a linear regression between the log frequencies for each sgRNA across time points 0, 2, 4, 6, 10, 12, and 14 (Supplementary Tables S5 and S6). In this mathematical definition, negC_rand_42 (wildtype) has a growth rate of zero. Before fitting, the time axis was rescaled to the number of generations by multiplying time (in hours) by the growth rate for the mixed population culture in the turbidostat. This was particularly important to enable comparisons between the glucose and glycerol environmental conditions. The mixed population growth rate under turbidostat conditions in glucose was 0.94 doublings h^−1^, in glycerol the growth rate was 0.53 doublings h^−1^. Importantly, the growth rate calculation was across all time points unless the total NGS counts for a given point dropped below 10, in which case that point was added to the fit, but no additional points were used. To ensure high data quality, fits with an *R*^2^ < 0.70 were removed from the analysis. However, *R*^2^ decreases significantly near slope = 0, so fits with a growth rate effect between −0.05 and 0.05 were not *R*^2^ filtered. Next, the q-test with a 95% confidence interval (CI) was used to filter out single internal replicates that deviated in growth rate from the remaining internal replicates. Lastly, sgRNAs without at least three internal replicate measurements after filtering were removed. The remaining replicates were averaged and the standard deviation and standard error of the mean were calculated using standard procedures.

**Figure 2.**
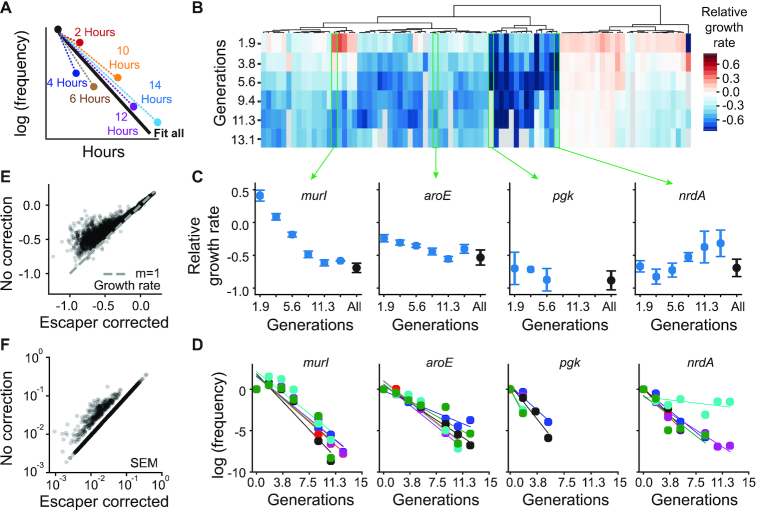
Time dependency of CRISPRi growth rate effects and escaper correction. (**A**) Schematic of the time dependent growth rate calculations. Time dependent effects of CRISPRi knockdown were calculated by linearly fitting the relationship between time point zero and every other time point individually. Colors are used to highlight the different fits used. (**B**) Heatmap of time dependent growth rate calculations calculated as described in A. Time dependent growth effects were clustered hierarchically along the time axis. (**C**) Growth effects over time for example genes from different clusters. The black time point is a linear fit using all the time points. The blue points indicate the growth at a given time point using only T0 and that time point to calculate growth rate. Error bars are SEM calculated from internal replicates. (**D**) Linear growth rate fits across all time points and internal replicates for the genes in (C). The slope of the line is relative growth rate. Different colored points relate to each of the six different internal replicate measurements. (**E**) Correlation plot comparing relative growth rates estimated by either using all time points and internal barcodes for escaper correction (x-axis), or a single time point (*t* = 14 h) and no internal barcodes. (**F**) Comparison of SEM across internal replicates with and without escaper correction.

To examine the time dependency of CRISPRi knockdown (Figure 2), we fit relative growth rates between two time points (*t* = 0 and one later time point) or all the time points. For this analysis, we did not impose a *R*^2^ or replicate filter, and the sequencing depth filter was set to greater than or equal to 10 sequencing counts. To assess the impact of escaper-correction on growth rate (in Figure 2E), we also calculated relative growth rates for all genes after pooling the counts across all six barcodes (i.e. across all six internal replicates), and considering only *t* = 0 and 14 h. The goal of this analysis was to mimic more standard analyses of relative enrichment. When fitting these growth rates, we did not impose a sequencing depth filter on the minimum number of counts per sgRNA. Relative growth rate effects in Supplementary Figure S1 were measured in a different CRISPRiSeq growth rate assay over a more limited set of sgRNAs and using three different replicate populations in different vials. These were incorporated into expression vectors without internal replicate barcode additions. These growth rates were used for consistency with qPCR measurements, which were also performed using the not barcoded plasmid.

**Figure 3.**
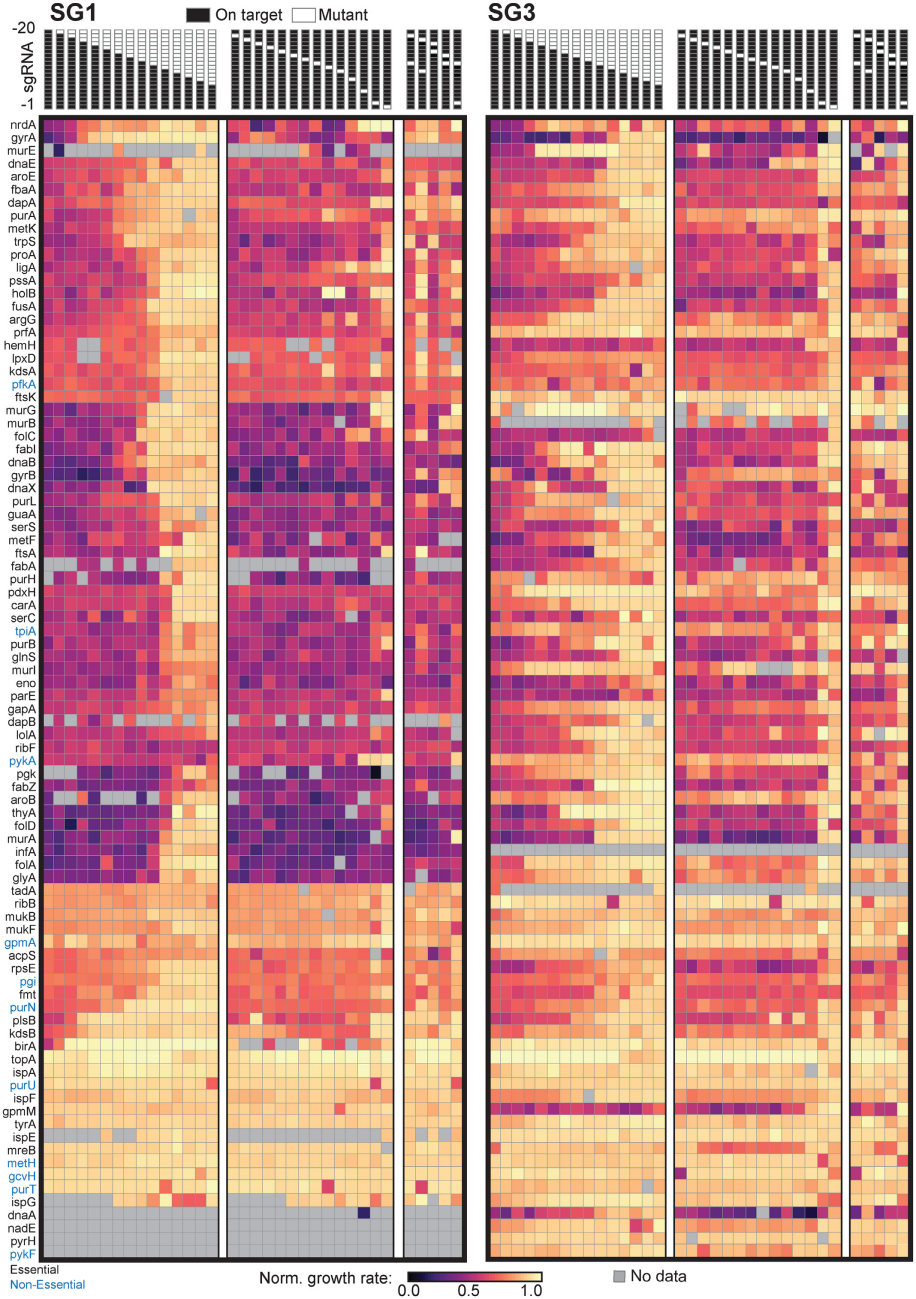
Heatmap of normalized growth rates for all sgRNAs in this study. Data was renormalized so that wildtype-like growth is 1 and no growth is 0 (methods). sgRNA design strategies are indicated at the top of the figure. Each box indicates a nucleotide in the sgRNA homology region. White boxes indicate a mutation to the complement nucleotide. Black boxes indicate that position is not mutated. Gene names are listed on the y-axis. Black gene names are essential based on previous reporting from the Keio collection, blue are nonessential. Grey squares indicate no data was collected for that specific sgRNA either because it could not be designed, it was not correctly synthesized, or data did not pass quality filters (Supplementary Figure S3).

After fitting relative growth rates, we rescaled them to a more intuitive scale, so that 0 indicates the maximum fitness defect (no growth) and 1 indicates wildtype-like (negC) sgRNA growth (Figure 3). To do this we selected a parent sgRNA that had an extremely severe effect on growth rate that also had six replicate measurements as an empirically observed growth rate ‘floor’. This was the *gyrB* SG2 parent sgRNA, which had a growth rate of −1.23 in M9-glucose. We added the absolute value of this growth rate to all variants and then divided by the absolute value of this growth rate. This rescaled negC_sgRNA_42 to one and the gyrB_SG2 parent sgRNA to zero.

### Calculating the number of resolvable growth effects

**Figure 4.**
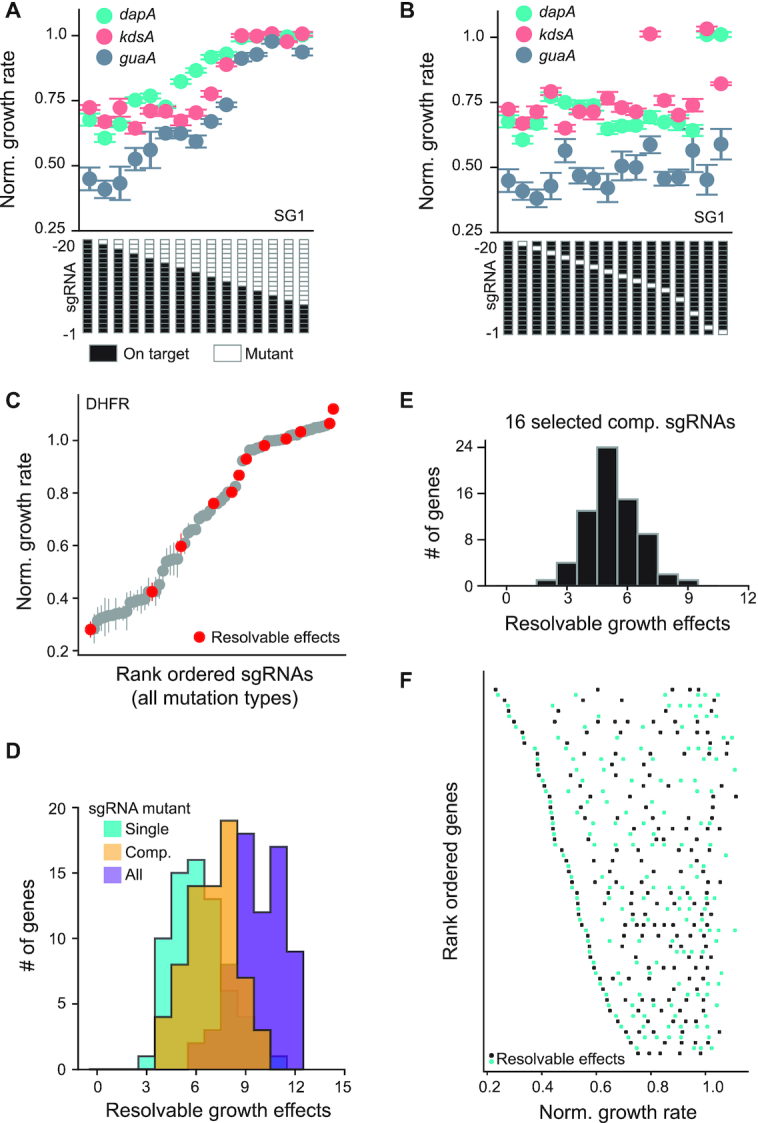
Design of a compact sgRNA library. (**A**) Relationship between the number of compounding mutations and normalized relative growth rate for three representative genes. Cartoons along the x-axis indicate the location of mutations. (**B**) Relationship between single mutations and normalized relative growth rate for the three genes shown in (A). (**C**) Example of statistically resolvable growth rate effects calculated over all sgRNAs targeting *folA* (the gene encoding DHFR). Statistically resolvable growth effects were calculated by rank ordering sgRNAs by mean growth rate effect and using *t*-tests with multiple hypothesis testing correction to identify significant steps (methods). (**D**) Histogram of the number of statistically resolvable normalized growth rates for single, compounding, and all designed sgRNAs. Cyan indicates single mutations, orange are compounding mutations, and purple are all mutations. (**E**) Histogram of the number of resolvable steps per gene when restricting the sgRNA library to 16 sgRNAs, comprising the on-target SG1 and SG3 sgRNAs plus mutations of these at positions 4–10. On average, genes showed five resolvable growth effects for this compact library. (**F**) Dot plot of resolvable steps for individual genes from the 16 sgRNA compact library (as in E). The x-axis contains the resolvable growth effects per gene. The y-axis displays genes rank ordered by their corresponding minimum normalized growth effect. Alternating cyan and black are only used to help the reader visualize the data.

To test different sgRNA mutation strategies, we calculated the number of statistically resolvable effects on growth per gene. We restricted this analysis to genes with both SG1 and SG3 titrating sgRNAs as well as at least one parent sgRNA that had a normalized growth rate effect of <0.75. The number of resolvable growth rate effects was calculated using Welch's *t*-test and a sequential goodness of fit (SGoF) multiple hypothesis testing correction (35). First, Welch's t-tests were performed for all possible pairs of sgRNAs targeting a given gene with an alpha (*P*-value) cutoff of 0.05. Significance values for all genes were pooled, and the SGoF multiple hypothesis testing correction was applied to control for family-wise error rate (metatest alpha = 0.05). Following SGoF, sgRNAs were separated by target gene and rank-ordered by their effect on growth rate. We determined the maximum number of resolvable effects on growth within each gene by finding the largest list of sgRNAs with mutually distinguishable effects on growth rate. Dynamic range and variance were maximized when there were multiple combinations of sgRNAs with the same number of resolvable effects. This analysis was performed separately for all compounding mutation sgRNAs, all single mutation sgRNAs, and all sgRNAs. We also used this strategy to calculate number of resolvable effects in the compact library (Figure 4D).

### Quantifying gene-by-environment growth effects

**Figure 5.**
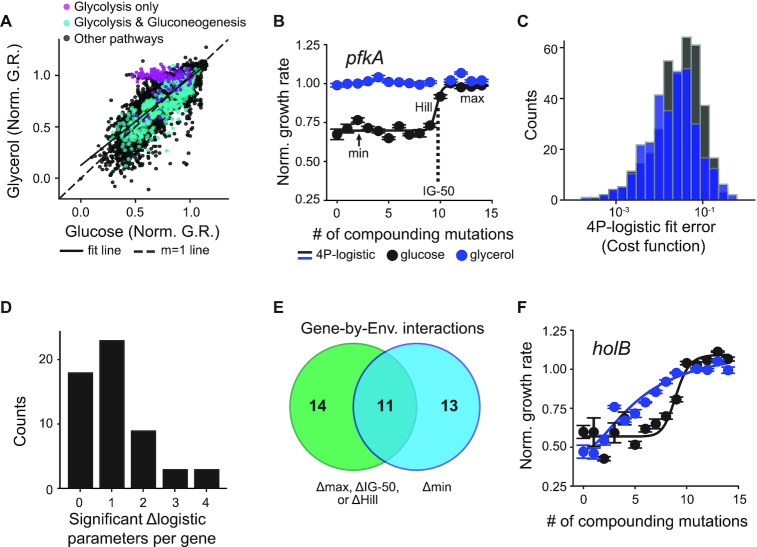
Comparison of sgRNA growth rate effects under glucose and glycerol carbon sources. (**A**) Correlation between glucose and glycerol normalized growth rates. Purple corresponds to *fbaA* and *pfkA*, two enzymes that are expected to have strong effects on growth rate in glucose but not glycerol. Teal corresponds to the remaining nine genes in glycolysis that are not expected to have differential effects. Black corresponds to all other non-glycolysis genes in our dataset. (**B**) Example fit of the 4-parameter logistic equation using phosphofructokinase isozyme 1 (*pfkA*). Blue points and associated line are from glycerol. Black points and line are from glucose. Fit parameters are medians from fitting all six internal replicates individually. Min, max, IG-50, and hill parameters are marked for reference. (**C**) 4-parameter logistic fit error (cost function) from SciPy_Optimize fitting algorithm for all logistic fits and internal replicates. Error corresponds with size of residuals. At least 8 points per replicate were required to fit logistic parameters. Logistic fits were made in at least one environment for 63/88 genes. (**D**) Number of logistic parameters showing a significant difference between glucose and glycerol conditions per gene. (**E**) Venn diagram of gene-by-environment interactions identified using the min parameter (blue), or any of the other parameters (green). (**F**) 4-parameter logistic fits for the *holB* gene in glucose (black) vs. glycerol (blue). The *holB* gene has significant differences in the IG-50 and max parameters.

Growth rate fitting and filtering was performed identically for measurements in glycerol and glucose conditions except only time points 0, 2, 6, 10, 14 were sequenced for the glycerol condition. We then used logistic fit parameters to compare effects between glycerol and glucose carbon sources. Focusing on the SG1 compounding mutation series, we fit a 4-parameter logistic function relating the number of sgRNA mutations to growth rate for each of the internal replicates, given a particular gene/environment combination. This resulted in parameters for the minimum (min) and maximum (max) growth rates, a Hill coefficient (Hill), and number of mutations at which the growth rate is half maximal (IG-50) (Figure 5, Supplementary Figures S8–S10). Fits were performed using the SciPy optimize.least_squares function with following constraints. First, the sgRNA with the fewest mutations (yet a still-measurable growth rate) was required to have a normalized relative growth rate of less than or equal to 0.75 in at least one of the two environments. Second, each replicate had to have at least eight sgRNAs with measured growth rate effects to be fit. Third, knockdown of a few genes had no effect on growth in one environment; therefore, the IG-50 and Hill coefficients could not be fit. To identify these, we averaged the growth effects of the three sgRNAs with the fewest number of mutations (called meanL) and also averaged effects of the three the sgRNAs with the most mutations (called meanH). If the differences between these two averages was less than 0.05, we reported the min parameter as meanL, the max parameter as meanH, and both the Hill coefficient and IG-50 were left empty. Fourth, the least_square algorithm requires a starting guess for each parameter. For these we guessed 6, 1, meanL, and meanH for IG-50, Hill, min, and max parameters respectively. Finally, we bounded the min, max and IG-50 parameters. Min and Max had to be between 0 and 1.15 and IG-50 had to be between 0 and 14. Bounds for the Hill coefficient were not justified by measurement parameters. For every gene, the median value of each fit parameter was used for plotting logistic curves in figures.

### Calculating gene-by-environment interactions

Gene-by-environment interactions were determined by comparing the difference in logistic fit parameters for each gene between environmental conditions. For a given gene/environment combination, we obtained up to six estimates for each logistic fit parameter (IG-50, Hill, min, and max), because we fit each internal replicate (plasmid barcode) separately. Welch's *t*-test was then used to evaluate the likelihood that a given parameter has the same mean in both environments; we took *P-value* < 0.05 as an initial threshold for significance. For genes which showed a sigmoidal titration curve in one condition and no titration in the other (e.g. pfkA), we assigned *P*-values of zero to both the IG50 and Hill coefficient parameters, following the logic that these are clearly significant differences. Following *P*-value calculation, multiple hypothesis testing for each parameter was corrected using SGoF (metatest alpha = 0.05) (35).

### Quantifying changes in gene expression with qPCR

CRISPRi effects on gene expression were quantified using qPCR. For these experiments, sgRNAs were expressed from plasmids that did not contain the replicate barcoding region or next-generation sequencing primer sites used in growth rate experiments. CRISPRi stains were grown in M9 minimal media and dCas9 was induced with 50 ng/ml ATc for 3–6 h before qPCR measurements. For each gene, dCas9 induction time was the same across all sgRNAs. After induction time, cells were immediately lysed with PureZOL reagent (BioRad, Cat#7326890) and RNA was extracted following the standard BioRad procedure with glycogen as an RNA carrier. qPCR was performed using the Luna Universal Syber reaction kit (NEB) in the a CFX384 Real-Time System. Every reaction was performed in technical triplicate. As done previously, the *hcaT* gene was used as a normalization control (36). Changes in mRNA concentration were calculated using the ΔΔC_t_ method, see Supplementary Table S2 for primer sequences.

## RESULTS

### Titratable sgRNA library design and construction

Our overall goal was to titrate the knockdown of specific genes by using mutated sgRNAs to produce small (but experimentally resolvable) stepwise changes in gene expression and growth rate. Bikard *et al.* previously showed that fluorescent protein abundance could be titrated by a series of sgRNAs with compounding mutations: complement mutations sequentially added to an sgRNA, beginning distal to the protospacer adjacent motif (PAM, Figure 1B). Taking this as a starting point, we constructed four series of compounding mutation sgRNAs targeting the essential genes *dapA* *dapB*, *serC*, and *purC*. Then, for all 44 of these sgRNAs, we measured the resulting CRISPRi effect on both growth rate and transcript abundance (by qPCR, Supplementary Figure S1). For *dapB*, the mutations produced titrated effects on both expression level and growth rate as desired (Supplementary Figure S1B and F). However, for the remaining three genes, the relationship was more step-like: at a particular number of mutations in the sgRNA (ranging between 10 and 12) transcript abundance dramatically changed from near wildtype to low abundance (10–20% of wildtype levels). Thus, we sought to examine how sgRNA mutations and location might be combined to systematically generate sgRNA series with titrated effects on both expression and growth.

We selected 88 genes important to *E. coli* growth as targets for testing mutational strategies and developing our approach (Figure 1A). Of these, 76 were previously reported essential in minimal media based on extremely limited growth or a complete inability to construct the knockout strain (26). The remaining 12 genes were selected to complete the genes encoding two central metabolic pathways: glycolysis and one-carbon folate metabolism. Overall, the selected genes represent 10 diverse cellular processes, are dispersed across the chromosome, and sample four orders of magnitude in protein copy number per cell (Figure 1A and Supplementary Figure S2) (27,28).

For each gene, we then constructed a series of 34–69 sgRNAs (Figure 1B). We began with three unique parent sgRNA constructs. Parent sgRNAs have no mutations to the sgRNA homology region and target the non-template strand. Parent SG1 and SG2 were designed to target the gene sequence as close as possible to the start codon without binding to overlapping regions. SG2 was intended to control for off-target binding effects in SG1, by identifying cases where the effects of SG1 and SG2 greatly diverge. SG3 was designed to bind at least 200 base pairs downstream of the start codon; we estimated these sgRNAs would yield intermediate knockdown effects because the efficiency of CRISPRi repression decreases with increasing distance to the start codon of the gene (17,19,20). Each sgRNA homology region was checked for potential off-target binding sites using a BLASTn search against the MG1655 *E. coli* genome. In ten cases, SG1, SG2 or SG3 could not be constructed because all possible sgRNAs targeting that region of the gene had potential off target binding sites; all three sgRNAs were identified for 78 of the 88 genes.

Both SG1 and SG3 were then further modified to generate a library of single, double, and compounding sgRNA mutant expression constructs (Figure 1B, Supplementary Table S1). Fourteen compounding sgRNAs were designed as in Bikard and colleagues by the sequential addition of complement mutations starting distal to the PAM (17,18). For the single mutations, eight positions from −20 to −13 of the sgRNA were selected following previous evidence that mutations at these sites might yield a range of effects on expression (and thus growth) (17,18,20). Additionally, we mutated positions −12, −11, −10, −8, −5, −2, and −1 to sample more severe disruptions to CRISPRi activity. Double mutations were similarly weighted towards mutations in the −20 to −13 range. The selected double mutations were at positions −19/−13, −18/−11, −17/−15, −14/−12, and −12/−2. All mutations were made to the complement DNA nucleotide to limit the potential for off-target binding. Additionally, 45 randomly scrambled sgRNA homology regions were included as non-targeting controls. Following library assembly, deep sequencing showed good coverage for nearly all 5927 constructs (Supplementary Figure S3 and methods).

### Growth rate measurements by deep sequencing with error-correcting barcodes

Following the thinking that reduced expression of essential genes should decrease growth rate, we measured the effect of CRISPRi knockdown on growth as a proxy for changes in expression. While this strategy does not inform on gene expression directly, it has two advantages. First, growth rates can be measured with a throughput and resolution that is more difficult to establish for gene expression measurements. Secondly, given that our ultimate goal is to study growth-expression relationships, we wish to bias our sgRNA designs towards those producing measurable (e.g. non-lethal) but varied growth rate effects. While we expect changes in growth rate to imply changes in expression level, the reverse need not be true.

To measure the growth rate effect for every sgRNA, we used a modified version of CRISPRiSeq (23–25). The general strategy behind CRISPRiSeq is to transform a pooled sgRNA library into a selection strain (here *E. coli* MG1655 containing a chromosomally-encoded dCas9), perform selection, and use next generation sequencing to estimate the frequency of each sgRNA construct in the population, both before and after selection (Figure 1C–E). We modified this general method to include three technical improvements. First, rather than growing transformed cell populations as a bulk culture, we grew the cells in a turbidostat. This device maintains the culture at a fixed optical density (OD) by sensing the OD and adjusting media dilution rate accordingly. This ensures that the culture remains in exponential phase throughout the experiment and allows for controlled environmental variation.

Secondly, rather than sequencing a single post-selection time point, we collected samples every 2 h over the course of 17 h, yielding a trajectory of relative allele frequency over time (Figure 1C, Supplementary Tables S3 and S4). Given the dynamics of dCas9 induction, mRNA degradation, protein dilution, and protein degradation, we reasoned that the growth rate effects of knockdown likely exhibit time dependence. To examine this, we fit relative growth rates between time point zero and every other time point for all SG1 parent sgRNAs (Figure 2A). Our rationale was that this would mimic the common practice of sequencing only a single post-selection time point, and provide a snapshot of what our experiment would look like at alternative endpoints. These were compared to the growth rate fit across all collected time points. Relative growth rates near zero indicate sgRNAs with an effect similar to the non-targeting control, while negative growth rates indicate deleterious effects on growth rate. We observed variation in the inferred growth rates across early time points for some, but not all, genes (Figure 2B). For instance, *murl* knockdown increased growth rate at early time points then decreased growth rate at later time points. In contrast, knockdown effects of *aroE* were nearly constant throughout the experiment (Figure 2C and D). After time point 10 hours, the measured growth rate stabilized for 87% of the 71 genes for which growth rate could be calculated at time point 10, 12, and 14 hours (Figure 2B–D). Thus, in the remaining analysis, we fit a single relative growth rate for each sgRNA as the slope over all seven time points (Supplementary Tables S5 and S6). We rationalized that this would help correct for any random fluctuations between time points and would reflect the stabilized growth rate attained at later times. However, we observed a few responses in which growth rate initially decreased (as expected for essential genes) then later increased (e.g. *nrdA*, Figure 2C and D). We suspected that such trajectories might reflect so-called ‘escapers’: loss-of-function mutations in either the sgRNA or dCas9 that reduce gene knockdown.

This brings us to the third methodological improvement of our approach. To facilitate detection of such escaper or suppressor mutants, we included an additional DNA barcode for error detection and correction during library cloning (Figure 1D). Specifically, we constructed the sgRNA library six times, in six plasmid backbones, each of which contains a unique DNA barcode. We refer to these six barcoded plasmids as internal replicates. By analyzing growth rate across all six internal replicates, we can detect and remove replicate constructs with anomalous growth. For example, in the growth rate trajectories for *nrdA*, we observed that one of the six barcodes (indicated by the cyan replicate) exhibits a relative growth rate similar to the non-targeting control (Figure 2D). Replicates like this were detected and removed during data processing by filtering on growth rate fit quality (*R*^2^), and using a *q*-test, producing escaper-corrected growth rates (Supplementary Figure S4A–C, Materials and Methods). In total, this removes ∼1% of the ∼36 000 replicate measurements.

Many sequencing-based CRISPRi experiments ignore escapers (under the assumption that they are rare and or negligibly impact population growth rates), and take only a single time point. While this requires less sequencing depth, and simplifies experimental design, we wondered how it might impact the estimated growth rates. To investigate this, we compared relative growth rates estimated from our sequencing data by either: (i) using escaper correction and including all seven time points or (ii) not applying escaper correction and considering only a single time point (*t* = 14 h). In the second case, growth rates were either the same or higher, indicating that in some cases escapers can obscure growth defects associated with gene knockdown (Figure 2E and Supplementary Figure S4B). Growth rate overestimation due to inferred escapers was especially severe for the slowest growing mutations. Overall, we identified 301 CRISPRi knockdowns (5% of the sgRNAs) wherein growth was significantly higher in the absence of escaper correction. Indeed, for one data point (SG3 targeting *serS*, mutation at −11), we observed a greater than 9-fold increase in the estimated growth rate when escapers were ignored. Removing escapers also improves the overall measurement error, as expected (Figure 2F). This indicates that both escaper correction and multiple time points are important to ensuring accurate relative growth rate measurements.

After removal of poorly fit relative growth rates (Supplementary Figure S4C and D), we observed that internal replicates were highly correlated (*R*^2^ = 0.8). Following escaper correction, the median measurement error across replicates was 9.7% (Supplementary Figure S5). Consistent with expectation, the growth rate effects for the SG1 and SG2 parent guides—both located near the start codon of the gene—were highly correlated, suggesting that off-target effects for particular sgRNAs do not play an outsized role (Supplementary Figure S5F). We also confirmed that the growth rate measurements inferred by sequencing of a mixed population were well-correlated to those measured by optical density over time in a plate reader for a sample of 28 sgRNAs (Supplementary Figure S5H). Taken together, these findings indicate that our CRISPRiSeq approach is yielding accurate, precise growth rate measurements with excellent throughput.

### The relationship between sgRNA location, sgRNA mutations, and the growth rate effect of gene knockdown

Next we wished to examine growth rate effects across the entire library. To facilitate this, we normalized all growth rates to a scale from zero (lethality) to one (wild-type like growth, methods) and plotted the results as a heatmap (Figure 3). We expected that parent sgRNAs (SG1, SG2, and SG3) targeting essential genes should result in large, measurable growth rate defects upon dCas9 induction—provided that transcription is effectively repressed. Consistent with this, we observed that the SG1 parent guides had an average normalized growth rate of 0.62 upon knockdown (Supplementary Figure S6A). As anticipated, SG3 parent guides were associated with more moderate effects on growth than SG1 parent guides, but still displayed an average normalized growth rate of 0.7 (Supplementary Figures S5G and S6B). The exception to the overall pattern of deleterious growth rate effects were 18 genes for which none of the parent sgRNAs decreased growth rate below 0.75. Twelve of these were previously reported essential in MOPS minimal media (*ftsK*, *nadE*, *ribB*, *ispE*, *ispF*, *ispG*, *ispA*, *tyrA*, *mukF*, *tadA*, *pyrH*, and *topA*) (26). For five of these genes (*ribB*, *ispA*, *mukF*, *tadA*, and *topA*), we verified that both the SG1 and SG3 parent sgRNAs yielded modest-to-no growth rate defect by independent measurements in a plate reader, and confirmed that both parent guides resulted in an expression knockdown by qPCR (Supplementary Figure S7). Other groups have also noted a range of growth rate defects associated with CRISPRi knockdown of essential genes (25). Potential (and non-exclusive) explanations for this include: (i) near-complete knockdown (approaching gene deletion) is necessary to produce a growth rate defect, (ii) the gene is required for exiting stationary phase but is not essential for exponential growth, (iii) the knockdown has polar effects and represses multiple downstream genes (i.e. within an operon), leading to a growth rescue or (iv) the original report of essentiality was somehow an experimental artifact. Notwithstanding, we observed that at least one of the parent sgRNAs yielded robust growth rate defects for 70 of the 88 genes in our test set. From this, we concluded that the majority of designed parent sgRNAs were working to effectively repress transcription.

Next, we examined the relationship between mutations to the sgRNA homology region and the growth rate effect of CRISPRi knockdown. While the mean growth rate effect for all sgRNAs in the library was deleterious (mean = 0.77), we observed a broad distribution of effect size (standard deviation = 0.22. Supplementary Figure S6C), indicating that our mutational approach was working to create sgRNAs with varied and subtle effects. We found that the compounding mutation sgRNA series exhibited a more intuitive relationship between mismatches and growth rates in comparison to the single mutation sgRNA series (Figures 3, 4A and B). The normalized growth rates for the compounding series varied in a near-monotonic fashion as mutations were added with increasing proximity to the PAM. In contrast, single and double mutations to the sgRNA showed less straightforward relationships to growth rate. For example, single mutations at position −1 and −2—mutations in the seed region that were expected to be extremely deleterious to CRISPRi activity—displayed a wide range of gene-dependent effects on normalized growth rate. Consistent with other reports, this suggests that many factors—including mutation location, mutation identity, sgRNA position, and even sequence context—all play a role in shaping knockdown effect (18,37,38). However, the compounding sgRNAs generally had a more intuitive and monotonic relationship with growth rate. Based on this, we sought to devise a simple and general set of empirical rules for generating libraries of titrating sgRNAs.

### A compact library design for titrating growth rate and expression

Originally, we designed 69 sgRNAs per gene to titrate gene expression. We wondered if a similar degree of titration could be achieved using significantly fewer compounding mutation sgRNAs. A compact library like this would be less expensive to synthesize, reduce sequencing depth requirements, and improve combinatorial scaling for future experiments targeting pairs (or more) of genes. So, given our data, how might we subsample the SG1 and SG3 guides to accomplish this? To define such a strategy, we considered 69 genes where at least one parent sgRNA (SG1, SG2 or SG3) yielded a normalized growth rate of 0.75 or less, with the rationale that these genes show strong growth defects upon knockdown. We then calculated the number of statistically resolvable growth rate effects per gene associated with each mutation strategy (either compounding or single mutations, Figure 4C). Across all sgRNAs (single, double, and compounding mutations of SG1, SG2, and SG3), we observe 6–13 resolvable steps per gene, with an average of 10 (Figure 4D). Considering single mutations only (32 sgRNAs), we observe 3–11 steps; similarly for the compounding mutation series (30 sgRNAs), we observe 4–10 steps (Figure 4D). Thus, the compounding mutation series capture a large proportion of the variation in growth rate with less than half of the total sgRNAs.

Given these findings, and considering the more intuitive relationship between compounding mutations and growth rate, we decided to focus on the compounding sgRNA series for compact library design. To select sgRNA mutations, we then examined the distribution of growth rate effects across all 69 genes for the compounding mutations at each position (Supplementary Figure S6D). Compounding mismatches at positions 4–10 aligned with the largest variation in growth rates. Accordingly, a library based on SG1 and SG3 that incorporates these seven mismatches plus the on-target guides (16 sgRNAs per gene) efficiently captures resolvable changes in growth rate in our data set (Figure 4E and F). In this compact library we see an average of five resolvable steps over 16 sgRNAs, indicating that ∼31% of the sgRNA library yields resolvable effects. For comparison, we observed an average of seven resolvable steps over the complete set of 30 compounding sgRNAs (∼23%), and an average of 10 resolvable steps over all 69 single, double, and compounding mutation sgRNAs (∼14%). Thus, subsampling specific compounding mutations at positions 4–10 provides a simple strategy for creating compact titrated sgRNA libraries. Extending this approach genome wide in *E. coli* would require ∼10^5^ sgRNAs; a scale readily accessible both by library construction and CRISPRiSeq technology. This approach now enables genome wide studies of the relationship between gene expression and growth rate.

### Mapping gene-by-environment interactions as a function of expression level

The environment plays a key role in modulating the relationship between gene expression and growth rate. Quantifying this effect is critical to understand how cells adapt to environmental stress, and can be used to discover and assign protein function (6,11). Because our experiments were performed in a multiplexed turbidostat, it was relatively straightforward to introduce well controlled environmental perturbations. We chose carbon source variation—changing between 0.4% glucose and 0.2% glycerol in M9 minimal media—as a well-characterized environmental perturbation for assessing the ability of titrated CRISPRiSeq to detect gene-by-environment interactions.

We repeated the CRISPRiSeq experiment with the complete library of sgRNAs under glycerol conditions at a subset of time points, and compared the resulting relative growth rates to those measured in glucose. To account for the overall slower growth rate in glycerol, we used the growth rate measured for the mixed population in the turbidostat (0.5 doublings h^−1^ in glycerol, 0.94 doublings h^−1^ in glucose) to rescale the time axis to units of generations (methods). Overall the relative growth rate effects were well correlated after this rescaling (*R*^2^ = 0.70) (Figure 5A). Consistent with known biology, we observed that knockdown of glycolytic genes which do not participate in gluconeogenesis (*pfkA* and *fbaA*) had severe growth effects in glucose but not glycerol (Figure 5B and Supplementary Figure S8). In addition, we observed relatively minor variations in growth effect between carbon sources for nine other enzymes (*pgi, tpiA, gapA, pgk, gpmA, gpmM, eno, pykA, and pykF*) that participate in both glycolysis and gluconeogenesis (Figure 5A). We note that the pyruvate kinase genes, *pykA* and *pykF*, play an important role in pyruvate production in both carbon sources, consistent with our observations, even though they are often considered as glycolysis genes (39,40). From these results, we concluded that CRISPRi knockdowns yielded comparable effects on gene expression in both environments, and that changes in growth rate between environments reflect gene-by-environment interactions.

While many high-throughput studies of gene-by-environment interactions focus on the growth rate effect of a single gene knockout or severe knockdown, titratable CRISPRi yields multiple measurements of growth rate under both environments. We thought that such titration curves might provide additional information beyond the limiting case of gene deletion, potentially exposing other types of interactions. To examine this, we focused on the compounding mutational series of SG1. We fit a 4-parameter logistic function describing the relationship between the number of compounding mutations and relative growth rate (Figure 5B). This fitting was performed for individual replicate measurements of 63 genes; these genes met the criteria that: (i) the sgRNA with the fewest mismatches showed a growth rate of less than 0.75 in at least one of the two environments and (ii) there were at least three replicates with 8 growth rate measurements (or more) per gene in both environments (methods). The resulting cost function indicated that the logistic fits provided a reasonable description of the data (Figure 5C). To find new gene-by-environment interactions that could not be identified using a single knockdown or knockout per gene, we looked for genes with no statistically significant difference in the minimal growth rate (‘min’ parameter), but significant differences in the IG-50, Hill, or max parameters (Supplementary Figure S8 and methods). Importantly, logistic fit parameters can only be compared between carbon sources for the same gene because parent sgRNAs targeting different genes may not have the same effects on gene expression. We identified significant changes in the logistic fit parameters between environments using Welch's t-test with sequential goodness of fit (SGoF) multiple hypothesis testing correction (35). In many cases, the environment modulates only one or two of the four logistic fit parameters (Figure 5D). Based on this analysis, we found 14 gene-by-environment interactions (37% of the total) that would not have been identified using a single knockdown per gene (Figure 5E, Supplementary Figures S9 and S10). Thus, our approach reveals a far more expansive set of environmental interactions than screening with knockouts alone.

As an example, consider *holB*, an essential gene that encodes the δ’ subunit of DNA polymerase III loading complex (Figure 5F). For this gene we did not detect a gene-by-environment interaction at the limit of minimum growth rate (maximum knockout effect). However, intermediate growth rate effects were much less severe in glycerol, leading to a significant difference in the IG-50. Thus, while *holB* is essential in both glucose and glycerol, it is coupled to the environment at intermediate expression levels. This gene-by-environment interaction is idiosyncratic across the DNA replication machinery: considering all eight DNA replication genes in our dataset, five showed an environmental interaction, with three of these only detectable using parameters other than minimal growth rate (Supplementary Figure S9). In addition to environmental interactions for genes in glycolysis and DNA replication, we also observed interactions with genes in numerous other cellular processes, including purine metabolism and peptidoglycan biosynthesis (Supplementary Figure S10). Taken together, this indicates that our data can pinpoint individual genes within diverse pathways that contribute to growth in a specific environment.

## DISCUSSION

In this work, we combined CRISPRiSeq with mutated sgRNAs to create a new approach for titrating gene expression and quantifying the resulting growth rate effects. By incorporating molecular barcodes for error correction and sampling multiple time points for growth rate estimation, our method unmasked deleterious effects of gene knockdown that would typically remain undetected. Moreover, we found that titrated variations in gene expression can expose a large number of additional gene-by-environment interactions missed by considering only a single maximal knockdown point. Given recent advances in highly parallelizable continuous culture devices (32,41), we imagine applying titratable CRISPRi to construct high-dimensional landscapes of growth rate given titrated perturbations in both gene expression and environment.

In addition to our work, several other recent studies have described high throughput approaches for generating intermediate perturbations in gene expression or activity. Together, these methods present an emerging suite of strategies for mapping the relationship between expression, catalytic activity, and growth rate in varied model organisms. For example, in *E. coli*, CRISPR-enabled trackable genome engineering (CREATE) combines sgRNAs with homologous repair cassettes to introduce mutations at nucleotide resolution on the scale of ∼10^4^ mutations (42). This approach can be used to engineer promoter libraries, explore the effects of mutation across a protein, or even introduce mutations across an entire pathway (43). However it is somewhat limited by editing efficiency (reported near 75%), and the resulting possibility of unedited WT escapers that can contribute to growth. In yeast, Bowman *et al.* recently showed that libraries of sgRNAs tiled across the promoter region can be combined with dCas9 fusions to Mxi1 (induction of heterochromatin formation) or VPR (recruitment of the mediator complex) to yield titrated down- and up-regulation of expression (44). In human cells, another recent study presented a strategy similar to our own, in which sgRNA knockdown strength was titrated using single and double mutations in the homology region in human cells (37). The authors measured growth phenotypes for mutated sgRNAs targeting genes in both Jurkat and K562 cells under a single environmental condition, confirming that titratable CRISPRi is readily generalized across organisms and cell types. Based on these data, a Convolutional Neural Net (CNN) regression model was trained to relate the sgRNA homology region sequence to growth rate phenotype. While this model presents an alternative approach to design compact sgRNA libraries, we suggest that compounding mutations present a more straightforward and robust strategy for creating gene expression titration curves. We also anticipate that all of these approaches could be enhanced using the barcoding and escaper correction techniques described here.

High throughput screens of gene essentiality, genetic interactions, and gene-by-environmental effects are often implemented with single genetic perturbations (e.g. total knockouts of the genes of interest) out of sheer technical necessity. Such screens form a cornerstone of modern genetics, and are highly productive in assigning membership of genes to biological processes and annotating genes of unknown function (6,11,45). However, these binary perturbations to gene activity limit our ability to construct quantitative, predictive models. Titratable CRISPRi now provides a path to more deeply characterize expression-to-phenotype relationships, and construct continuous models relating expression to cellular phenotype. By combining pairs (or more) of sgRNAs targeting different genes, one might examine the dependence of epistatic relationships, such as synthetic lethality, on knockdown strength. We envision that our approach can be applied to optimize or tune biochemical pathways, uncover new therapeutic targets, and understand how variation in expression influences drug resistance. Perhaps most fundamentally, these experiments now provide a platform for training and deeply testing predictive models of cell growth given variation in gene expression levels.

## DATA AVAILABILITY

All codes for sgRNA design and next generation sequencing data analysis are available on github: https://github.com/reynoldsk/titratableCRISPRi. The codes are implemented in Python, provided as Jupyter notebooks, and are sufficient to recreate all figures in this work. The sgRNA read counts and associated calculated growth rates are supplied as supplemental tables.

## Supplementary Material

gkaa1073_Supplemental_Files
