# Supplementary material for: A simplified strategy for titrating gene expression reveals new relationships between genotype, environment, and bacterial growth
Source: Nucleic Acids Res. 2020 Nov 22;49(1):e6. doi: 10.1093/nar/gkaa1073 (PMC7797047; doi:10.1093/nar/gkaa1073)
Supplement: gkaa1073_Supplemental_Files [file gkaa1073_supplemental_files.zip › 201002-Mathis-Supplemental.docx]

**Supplementary Material for**

**A simplified strategy for titrating gene expression reveals new relationships between genotype, environment and bacterial growth**

Andrew D. Mathis^1^, Ryan Otto^1^ and Kimberly A. Reynolds^1,2*^

^1^ The Green Center for Systems Biology and ^2^Department of Biophysics, The University of Texas Southwestern, Dallas, TX, 75390, USA

This PDF file includes:

Figures S1 to S10

Descriptions of Tables S1 to S6

**Figure S1: Compounding sgRNA effects on gene expression and growth rate. A-D.** Relative gene expression was quantified using qPCR for arrays of compounding sgRNA mutations for the *dapA, dapB, serC, and purC* genes. qPCR error bars are from technical replicates. These genes are all essential. Compounding mutations were serially added starting at position -20. Mutations were always to the complement nucleotide. **E-H.** Correlation between gene expression and growth rate effects measured using CRISPRiSeq. Growth rate error bars are from three replicate experimental vials (bacterial culture populations). Growth rate is normalized to a negative control sgRNA with no homology region.

**Figure S2: Additional properties of the 88 genes selected for CRISPRi targeting.**

**A.** Chromosomal locations on the MG1655 *E. coli* genome (NC000913.3) of the 88 selected genes. **B.** Histogram of estimated protein copy numbers for the 88 selected genes. Estimations based on (27).

**Figure S3: sgRNA library completeness.** Next-generation sequencing counts for the assembled sgRNA library; data are shown for a single representative internal replicate with molecular barcode ATCATG. Grey indicates sgRNAs that could not be designed because all possible sgRNAs targeting that region of the gene had high potential for off-target effects. White indicates that the sgRNA was designed, but not found in the synthesized library. sgRNA mutation types are labeled on the bottom of the plot.

**Figure S4: Escaper correction and data quality filters. A.** An example of identifying and removing an escaper mutation using a q-test. This is a histogram of growth rates for internal replicate measurements of a *ribF* gene knockdown. The escaper mutation (highlighted in red) is identified by a q-test and removed before calculating mean relative growth rate. **B.** The effect of escaper mutations on overall relative growth rate measurements. The escaper effect was calculated by taking the difference between escaper corrected and non-corrected relative growth rates (see also Figure 2E). The negative sign indicates a lower growth rate for the escaper corrected measurement. Non-corrected growth rates were calculated by pooling counts across all six internal replicates (ignoring molecular barcodes) and only considering timepoints t=0 and t=14 hours. **C.** Histogram of R^2^ values for the linear regression of relative growth rates across all sgRNAs and internal replicates. Relative growth rates with associated R^2^ values less than 0.7 were excluded from downstream calculations; with the exception that no filtering was performed for small relative growth rate effects between -0.05 and 0.05. **D.** Histogram of internal replicates per sgRNA. At least three internal replicate barcode measurements had to be made per sgRNA to be included in downstream analyses.

**Figure S5: CRISPRiSeq data quality. A-C.** Correlations between internal replicate growth rate measurements. **D.** Heatmap of R^2^ values in all-by-all internal replicate correlations. **E.** Relationship between internal replicate growth rate and standard error of the mean. **F.** Growth rate correlation between SG1 and SG2 parent sgRNAs. **G.** Growth rate correlation between SG1 and SG3 parent sgRNAs. **H-I.** Correlation between relative growth rates measured for isolated cultures in 96-well plates and measured by CRISPRiSeq. Error bars along the x-axis represent SEM across three technical replicates, error bars along the y-axis represent SEM across the internal replicates. A black line indicates the diagonal.

**Figure S6: CRISPRi sgRNA library effects on growth rate. A-B.** Histogram of normalized relative growth rates for SG1 and SG3 parent sgRNAs, respectively. **C.** Histogram of normalized growth rates for all sgRNAs. **D**. Box and whisker plots indicating the distribution of growth rates for a given compounding mutation across all 88 genes. The data were renormalized such that a growth rate of zero is equivalent to the SG1 parent (non-mutated) sgRNA growth effect. Growth rates were organized by SG1 (top plot, red) or SG3 (bottom plot, grey). Arrows indicate the 16 sgRNAs that were included in the compact library in Figure 4E-F. These were selected by looking for changes in the mean effect on the box plot by eye.

**Figure S7: CRISPRi sgRNAs targeting essential genes, but with limited growth defects. A.** Comparison of relative growth rates measured by CRISPRiSeq (in a mixed population) or as individual growth curves in a 96 well plate. Color indicates gene identity, the two points for each gene indicate knockdowns with either the SG1 or SG3 parent sgRNA. Error bars represent SEM across triplicate measurements. **B.** Relative gene expression as determined by qPCR, following CRISPRi induction for targeted SG1 and SG3 knockdowns. Each color represents one gene, and maps to panel A. All measurements are relative to negC (a non-targeting sgRNA) and normalized against the *hcaT* housekeeping gene. Error bars represent SEM across triplicate measurements.

**
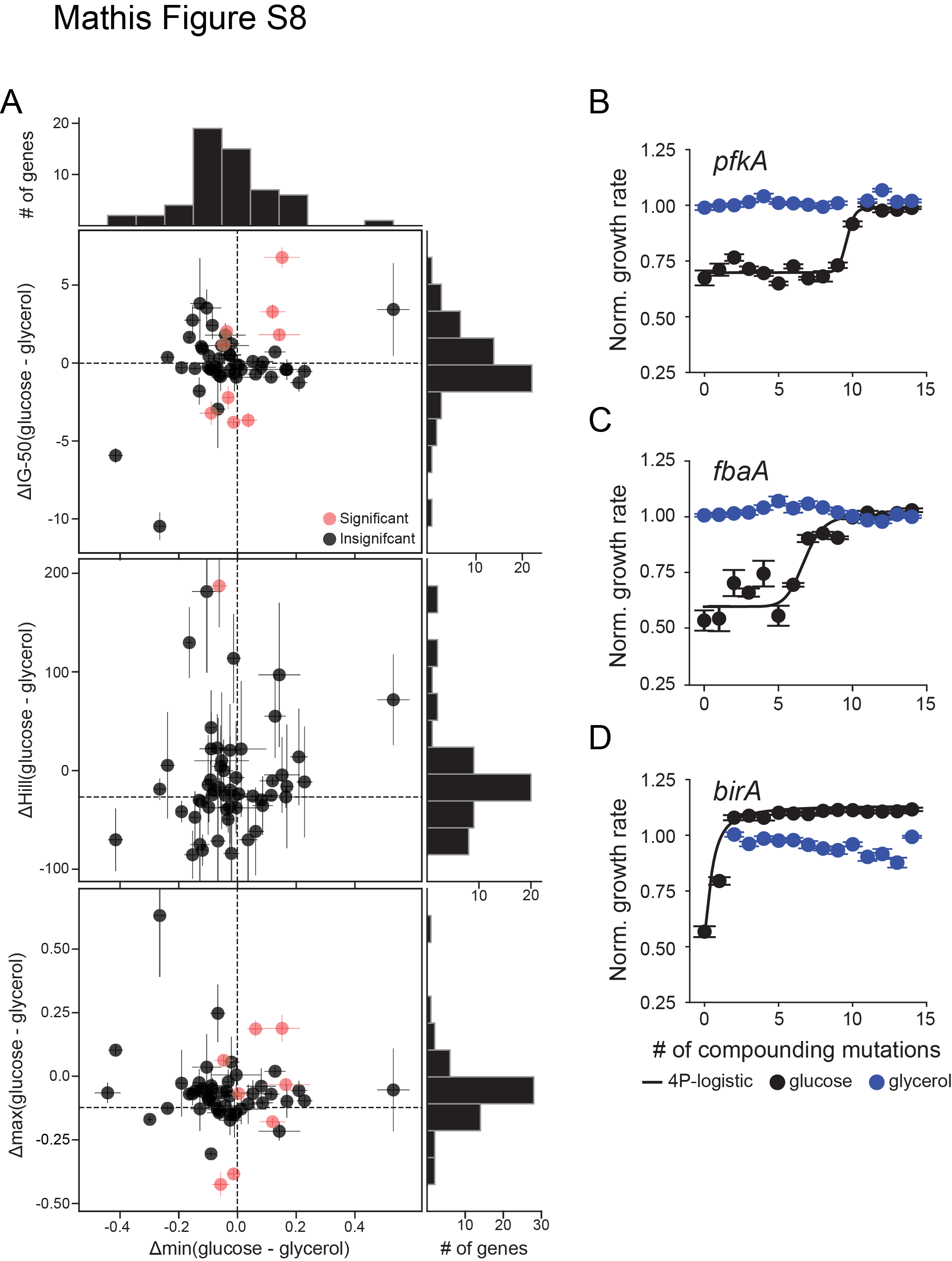
**

**Figure S8: Comparison of sgRNA growth rate effects under glucose and glycerol carbon sources. A.** Scatter plot of changes in min, max, IG-50, and Hill parameters. The x-axis shows differences between the min parameters in glucose and glycerol. The y-axis displays differences between the IG-50, Hill, and max parameters (top, middle and bottom plots respectively); associated histograms indicate the distribution of differences for each parameter. In red are genes that had a significant difference in Hill or IG-50 parameters, but no significant difference in the min parameter. **B-D**. Gene-by-environment titration curves for three genes with significant differences between environments that could not be shown in panel (A) because they lack a titration curve in glycerol.

**Figure S9: Growth rate titration curves for genes involved in DNA replication. A-H** Normalized relative growth rates vs number of compounding mutations for SG1 sgRNA variants targeting eight genes involved in DNA replication. Data collected in glucose and glycerol are shown in black and blue respectively. Points indicate the mean growth rate across internal replicates, and error bars represent the SEM. The smooth curves indicate the best fit logistic regression. A box at the bottom right lists logistic regression parameters that show a statistically significant difference between glucose and glycerol environments.

**Figure S10: Growth rate titration curves for genes with significant changes in IG-50, growth rate max, and Hill coefficient but not growth rate min. A-K** These eleven genes show gene-by-environment interactions undetected at the limit of maximal knockdown. DNA replication genes show in Supplementary Figure S9 are not repeated here. Scatter plots show normalized relative growth rates vs number of compounding mutations for SG1 sgRNA variants. Data collected in glucose and glycerol are shown in black and blue respectively. Points indicate the mean growth rate across internal replicates, and error bars represent the SEM. The smooth curves indicate the best fit logistic regression. A box at the bottom right lists logistic regression parameters that show a statistically significant difference between glucose and glycerol environments.

**Tables**

Table S1: sgRNA library design. A list of all homology region sequences, annotated by gene, mutation type, and mutation location.

Table S2: Primers

Table S3: Next-generation sequencing counts for each sgRNA at each timepoint in glucose condition

Table S4: Next-generation sequencing counts for each sgRNA at each timepoint in glycerol condition

Table S5: CRISPRi growth rate effect and corresponding measurement errors for glucose condition

Table S6: CRISPRi growth rate effect and corresponding measurement errors for glycerol condition
